# NPK Accumulation, Physiology, and Production of Sour Passion Fruit under Salt Stress Irrigated with Brackish Water in the Phenological Stages and K Fertilization

**DOI:** 10.3390/plants12071573

**Published:** 2023-04-06

**Authors:** Geovani Soares de Lima, André Alisson Rodrigues da Silva, Rafaela Aparecida Frazão Torres, Lauriane Almeida dos Anjos Soares, Hans Raj Gheyi, Francisco Alves da Silva, Reginaldo Gomes Nobre, Carlos Alberto Vieira de Azevedo, Kilson Pinheiro Lopes, Lúcia Helena Garófalo Chaves, Vera Lúcia Antunes de Lima

**Affiliations:** 1 Post Graduate Program in Agricultural Engineering, Federal University of Campina Grande, Campina Grande 58430-380, Brazil; 2 Post Graduate Program in Tropical Horticulture, Federal University of Campina Grande, Pombal 58840-000, Brazil; 3 Post Graduate Program in Soil and Water Management, Federal Rural University of the Semi-Arid, Caraúbas 59780-000, Brazil

**Keywords:** *Passiflora edulis*, water scarcity, osmotic regulation

## Abstract

This research aimed to evaluate the effects of salt stress, varying the phenological stages, and K fertilization on NPK concentrations, physiology, and production of *Passiflora edulis* Sims. The research was carried out at the University Farm of São Domingos, Paraíba, Brazil, using a randomized block design with a 6 × 2 factorial arrangement. Six irrigation strategies were evaluated (use of low electrical conductivity water (0.3 dS m^−1^) during all stages of development and application of high-salinity water (4.0 dS m^−1^) in the following stages: vegetative, flowering, fruiting, successively in the vegetative/flowering, and vegetative/fruiting stages) and two potassium levels (207 and 345 g K_2_O per plant), with four replications and three plants per plot. The leaf concentrations of N, P, and K in the sour passion fruit plants found in the present study were below the optimal levels reported in the literature, regardless of the development stage and the cultivation cycle. The relative water content, stomatal conductance, and photosynthesis were reduced by salt stress in the first cycle. However, in the second cycle, irrigation with 4.0 dS m^−1^ in the vegetative/flowering stages increased the CO_2_ assimilation rate. Passion fruit is sensitive to salt stress in the vegetative/flowering stages of the first cycle. In the second cycle, salt stress in the fruiting stage resulted in higher production per plant.

## 1. Introduction

*Passiflora edulis* Sims stands out as one of the main fruit species due to its high nutritive value, excellent organoleptic features, and significant economic potential as fresh fruit or in the agroindustry [1]. Although the northeastern semi-arid region has the potential for the increased cultivation of this fruit species, the occurrence of high temperatures and the irregular distribution of rainfall associated with intense evaporation throughout the year are limiting factors for irrigated fruit cultivation [2]. In this region, it is common to find water sources with high levels of dissolved salts, standing out as one of the abiotic stresses that promote osmotic and ionic changes in plants [3] when used to irrigate orchards.

Salts in the root zone cause osmotic stress, ion toxicity, nutrient imbalance, and water deficit, inducing a decrease in water and nutrient uptake by plants. Furthermore, excessive ion concentrations in the soil solution also damage photosynthetically active leaves and can result in chlorosis and early senescence, thus negatively affecting photosynthetic efficiency and production [4]. In this context, several studies have been carried out evaluating the effects of irrigation with saline water in the passion fruit crop, highlighting changes in gas exchange [5], photochemical efficiency [6], synthesis of photosynthetic pigments [7], and membrane instability [8], as well as a reduction in production components [9,10] and post-harvest quality [11]. However, these studies are restricted to the use of water with different levels of salinity throughout the plant development cycle, thus requiring further research, especially to assess the effects of salt stress on the accumulation of NPK, physiology, and production of sour passion fruit plants of the cultivar BRS GA1, one of the most commonly grown in northeast Brazil, at varying phenological stages.

It is important to highlight that the intensity of salt stress effects depends on the species, genotype, duration of exposure, fertilization, irrigation management, and development stage [12], in addition to stress tolerance mechanisms, including the maintenance of ion homeostasis and the osmotic balance and the elimination of reactive oxygen species [13]. Thus, the use of water with a high concentration of salts in the phenological phase(s) in which the crop expresses tolerance is a promising alternative for reducing the effects of salt stress [14,15]. Soares et al. [16] observed that salt stress in the initial stages leads to earlier flowering in cotton and does not compromise its production. Lima et al. [17] found that salt stress in the vegetative, flowering, and fruiting stages is a promising strategy for sesame cultivation in the semi-arid region of Brazil. Silva et al. [18], in a study evaluating the production and post-harvest quality of mini-watermelon fruits, saline water irrigation management strategies, and potassium fertilization, concluded that irrigation with water of 4.0 dS m^−1^ in the flowering and fruit maturation stages is a promising strategy for cultivation, since it does not compromise the production, and fertilization with 50% of the K_2_O recommendation can be used in the cultivation of mini-watermelon without losses in production.

Thus, the use of saline water in the phenological stage(s) in which the crop has higher tolerance is a promising irrigation strategy, as it allows minimizing the effects of salt stress on plants and reduces changes in the physical-chemical attributes of the soil due to the formation of saline and sodic soils.

Another strategy capable of mitigating salt stress in plants is potassium fertilization [12,13,14,15]. Potassium is involved in almost all plant physiological processes that require water. These processes include stomatal regulation, photoassimilate transport, enzyme activation, and heliotropic movements of leaves. Potassium also assists in water transport, translocation of mineral compounds to the entire plant through the xylem, and maintenance of ion homeostasis and osmotic balance [19]. Under salt stress conditions, Munir et al. [20] observed that high K^+^ levels improved the concentrations of antioxidant enzymes and the morphophysiological attributes of the plants. From this perspective, potassium fertilization could be an alternative means to maintain ion homeostasis, compensate for the negative charges of the macromolecules, maintain electroneutrality, and establish cell turgor and volume [21].

In this context, several studies have been carried out evaluating the effects of irrigation with saline water in the passion fruit crop, highlighting changes in gas exchange [5,22], photochemical efficiency [6], synthesis of photosynthetic pigments [7], and membrane instability [8], as well as a reduction in production components [9,10] and post-harvest quality [11]. However, these studies are restricted to the use of water with different levels of electrical conductivity throughout the plant development cycle, thus requiring further research, especially to assess the concentrations of NPK, physiology, and production of *Passiflora edulis* Sims, cultivar BRS GA1, grown under salt stress in the phenological stages and fertilization with K.

The hypothesis of this study is that the sensitivity and/or tolerance of passion fruit to salt stress varies with the development stage of the crop and that the intensity of salt stress effects on NPK accumulation, physiology and production can be attenuated by potassium fertilization through its function in osmoregulation, enzymatic activation, and oxidative protection. Thus, it is imperative to conduct new studies aimed at identifying the stage of the development cycle of BRS GA1 sour passion fruit in which it is sensitive and/or tolerant to water salinity, as well as a dose of potassium capable of mitigating the intensity of salt stress as an alternative for production in areas with qualitative and quantitative scarcity of water resources, a situation found in semi-arid areas of northeastern Brazil.

In this scenario, the aim of this study was to evaluate the NPK concentrations, physiology, and production of *Passiflora edulis* Sims under salt stress in the phenological stages and fertilization with K.

## 2. Results

The ISBW × KD significantly affected the leaf concentrations of nitrogen and potassium of *Passiflora edulis* Sims in the first and second cycle. The brackish water irrigation strategies significantly influenced all studied variables in the first cycle. In the second cycle, the ISBW had a significant effect on all variables except for the leaf P concentration. The potassium levels, in turn, significantly interfered with the nitrogen and potassium concentrations in the first cycle; in the second cycle, the KD significantly affected the K concentrations in leaves (Table 1).

**Figure 1:**
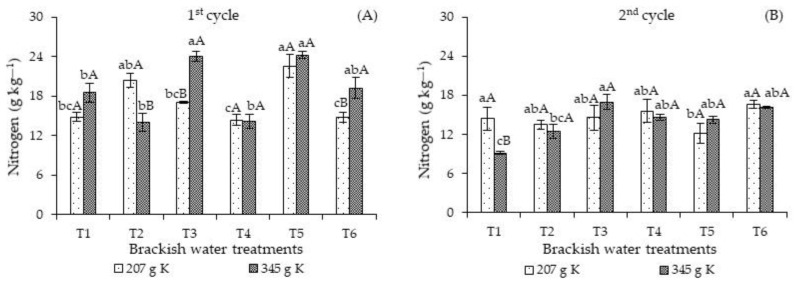
Leaf nitrogen concentrations of *Passiflora edulis* Sims as a function of the interaction ISBW × KD in the first (**A**) and second (**B**) cycle. Standard error of the mean (*n* = 4); Means followed by different lowercase letters indicate a significant difference between the irrigation strategies with brackish water for the same KD (*p* ≤ 0.05), and different uppercase letters indicate a significant difference between potassium levels for the same irrigation strategy. T1—irrigation with low-salinity water during the entire cultivation cycle; salt stress in the following stages: T2—vegetative; T3—flowering; T4—fruiting; T5—vegetative and flowering; T6—vegetative and fruiting.

In the first cycle (Figure 1A), the leaf nitrogen concentrations in plants subjected to the T3 and T5 strategies were higher than those found in the other strategies (T1, T2, and T4) when receiving 345 g of K_2_O. On the other hand, fertilization with 207 g of K_2_O resulted in the lowest N concentrations in plants cultivated under the T4 and T6 strategies compared to those subjected to salt stress in T2 and T5 strategies. When decomposing the effect of the potassium levels in each strategy, the N concentrations of plants subjected to the ECw of 4.0 dS m^−1^ in the vegetative stage and fertilization with 207 g of K_2_O were superior to those of plants that received 345 g K_2_O.

Despite the increase in the N concentrations, especially in plants fertilized with 345 g K_2_O and irrigated with high-salinity water in T3, T5, and T6, the values obtained (24.07 and 19.21 g kg^−1^) are considered insufficient for the adequate nutrition of *Passiflora edulis* Sims, which should range from 40 to 50 g kg^−1^ [23]. Carvalho [24] evaluated the effects of nitrogen fertilization, irrigation, and sampling time on the leaf nutrient concentrations of *Passiflora edulis f. flavicarpa Deg.* and observed that the leaf N concentrations were below the range considered adequate for the crop during the flowering and fruiting peaks. However, the plants did not show any nutritional deficiency symptoms.

For the second cycle (Figure 1B), plants under salt stress in T3, T4, T5, and T6 and fertilized with 345 g K_2_O obtained higher N concentrations than plants that received T1 during the entire cultivation cycle and high-salinity water in T2. On the other hand, treatments T2 and T4 and fertilization with 207 g K_2_O resulted in a higher N concentrations, differing significantly only from the T5 strategy. When analyzing the effects of the potassium levels in each irrigation strategy, the N concentrations of plants fertilized with 207 g K_2_O differed significantly only when irrigated with the lowest ECw water (T1) during the entire cycle. There were no significant differences in the remaining irrigation strategies regardless of the potassium level. When comparing the two cycles, the leaf N concentrations of the plants decreased markedly in the second cycle compared to the first.

**Figure 2:**
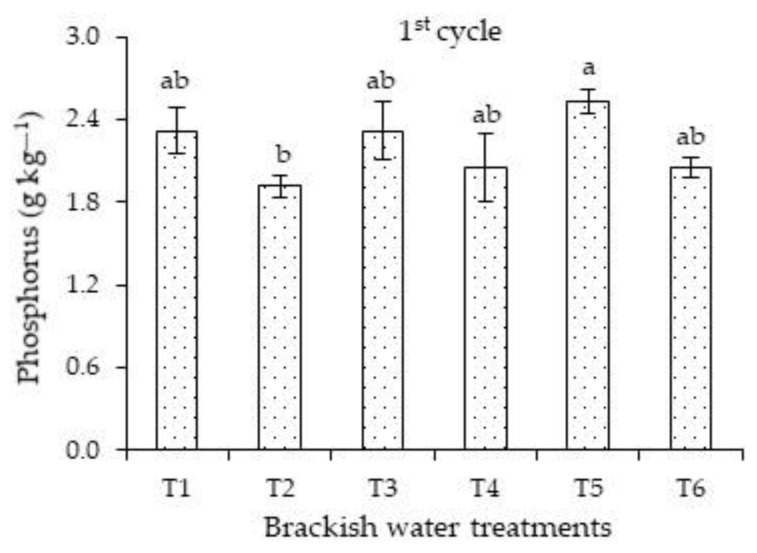
Leaf concentrations of phosphorus of *Passiflora edulis* Sims as a function of salt stress, varying the phenological stages in the first cycle. Standard error of the mean (*n* = 4); Means followed by different letters indicate a significant difference between treatments. T1—irrigation with low-salinity water during the entire cultivation cycle; salt stress in the following stages: T2—vegetative; T3—flowering; T4—fruiting; T5—vegetative and flowering; T6—vegetative and fruiting.

Concerning the effects of salt stress on the phosphorus concentrations of the first cycle, plants irrigated with high ECw successively in the vegetative and flowering stages differed significantly only from those cultivated under salt stress in the vegetative stage (Figure 2). There were no significant differences when comparing the concentrations of plants subjected strategies T1, T3, T4, T5, and T6.

**Figure 3:**
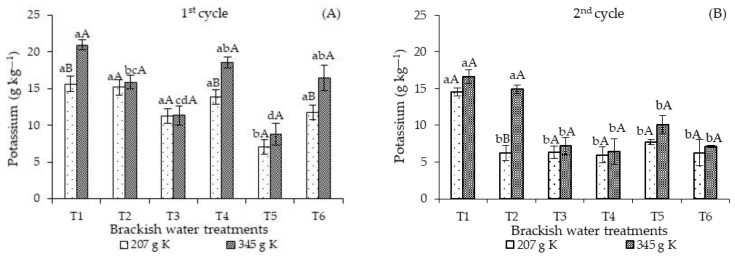
Leaf K concentrations of *Passiflora edulis* Sims as a function of the interaction ISBW × KD in the first (**A**) and second (**B**) cycle. Standard error of the mean (*n* = 4); Means followed by different lowercase letters indicate a significant difference between irrigation strategies with brackish water for the same KD (*p* ≤ 0.05), and different uppercase letters indicate a significant difference between potassium levels for the same irrigation strategy. T1—irrigation with low-salinity water during the entire cultivation cycle; salt stress in the following stages: T2—vegetative; T3—flowering; T4—fruiting; T5—vegetative and flowering; T6—vegetative and fruiting.

The potassium concentrations of sour passion fruit plants fertilized with 207 g of K_2_O and continuously irrigated with high-salinity water in T5 decreased significantly compared to those found in plants subjected to the other irrigation strategies (T1, T2, T3, T4, and T6) in the first cultivation cycle (Figure 3A). Also, there were no significant differences when comparing the control treatment (T1) with the T1, T3, T4, and T6 strategies. Furthermore, fertilization with 345 g of K_2_O promoted the highest leaf concentrations of potassium in plants subjected to the T1, T4, and T6 strategies. There were significant differences in the potassium concentrations of plants subjected to T2, T3, and T5 compared to the other strategies, T1, T4, and T6. When analyzing KD in each strategy, a clear superiority of the leaf K concentrations in plants that received 345 g K_2_O in T1, T4, and T6 was observed.

Regardless of the irrigation strategy and the cultivation cycles, the leaf concentrations of N, P, and K obtained in this study are below the range considered adequate [23]. However, it should be noted that fertilization management followed the recommendations of [25] and the plants during the crop cycle did not show any deficiency symptoms. Soares et al. [26] studied the effects of salinity on the early development of sour passion fruit and also observed that potassium uptake was reduced even at low salinity. According to these authors, this reduction occurred as a function of the loss in the selective absorption capacity of ions by the plasmalemma.

**Figure 4:**
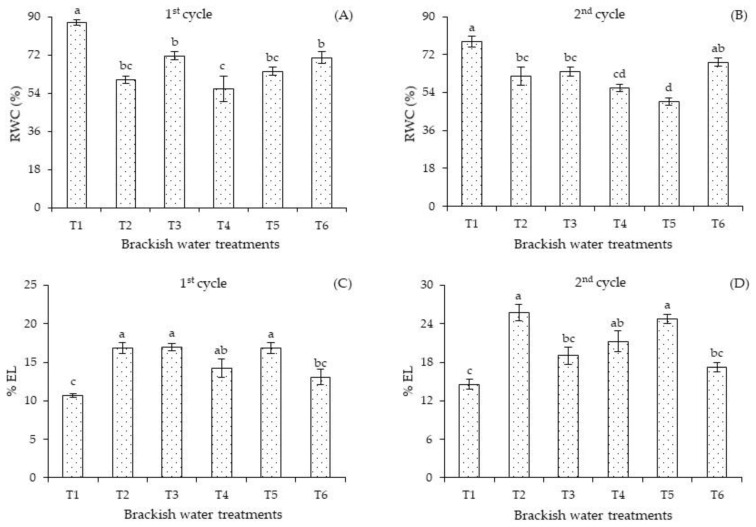
Relative water content—RWC (**A**,**B**) and electrolyte leakage—%EL (**C**,**D**) in *Passiflora edulis* Sims as a function of salt stress, varying the phenological stages in the first (240 DAT) and second (445 DAT) cycles. Standard error of the mean (*n* = 8); Means followed by different letters indicate a significant difference between treatments. T1—irrigation with low-salinity water during the entire cultivation cycle; salt stress in the following stages: T2—vegetative; T3—flowering; T4—fruiting; T5—vegetative and flowering; T6—vegetative and fruiting.

The ISBW × KD interaction also influenced the leaf K concentrations in the second cycle (Figure 3B). Fertilization with 207 g of K_2_O promoted the highest leaf K concentrations in plants subjected to T1 during the entire cultivation cycle, differing significantly from the other strategies. On the other hand, fertilization with 345 g K_2_O resulted in the highest K concentrations in plants cultivated under the T1 strategy and salt stress in the vegetative stage (T2), surpassing the values observed in the other irrigation strategies with brackish water. When analyzing the effects of potassium levels in each irrigation strategy (Figure 4B), significant differences were observed only in the K concentrations of T2, with plants fertilized with 345 g K_2_O attaining the highest values.

There was a significant effect of the irrigation strategies with brackish water on all variables measured in the first and second cycle (Table 2). However, the KD and the ISBW × KD interaction did not significantly influence any of the variables measured in the first and second cycle.

In the first cycle, the relative water content (Figure 4A) of plants subjected to T1 surpassed the values of plants that received high-salinity water in the other irrigation strategies (T2, T3, T4, T5, and T6). When comparing the RWC of plants subjected to water stress in the different phenological stages, significant differences were observed between T3 and T6 compared to T4. However, there were no significant differences in the RWC of plants subjected to salt stress in T2, T3, T5, and T6.

In the second cycle, the relative water content (Figure 4B) of plants cultivated under T1 differed significantly from that of plants that received salt stress in the T2, T3, T4, and T5 strategies. Plants subjected to continuous salt stress in T5 and T4 obtained the lowest RWC values, possibly due to water uptake restrictions.

Electrolyte leakage of plants subjected to salt stress in T2, T3, and T5 differed significantly compared to those grown under the T1 and T6 strategies in the first cultivation cycle (Figure 4C). No significant effect was observed when comparing the T2, T3, T4, and T6 strategies. In the second cultivation cycle (Figure 4D), irrigation with 4.0 dS m^−1^ promoted the highest electrolyte leakage of plants under the T2 and T5 strategies, differing significantly from those that received T1, T3, and T6. Similar to the first cycle (Figure 4A), irrigation with ECw of 1.3 dS m^−1^ during the entire cycle (T1) and the ECw of 4.0 dS m^−1^ in T6 resulted in a lower %EL. The reduction in %EL in the T6 strategy indicates the recovery potential of plants subjected to salt stress since, from the end of the vegetative stage to the beginning of the fruiting stage, the plants were irrigated for 19 days with the ECw of 1.3 dS m^−1^. Lima et al. [27] studied *Passiflora edulis* Sims plants irrigated with different cationic natures and observed that the water with the ECw of 3.0 dS m^−1^ containing Na^+^ and Na^+^ + Ca^2+^ increased electrolyte leakage in the leaf tissues.

**Figure 5:**
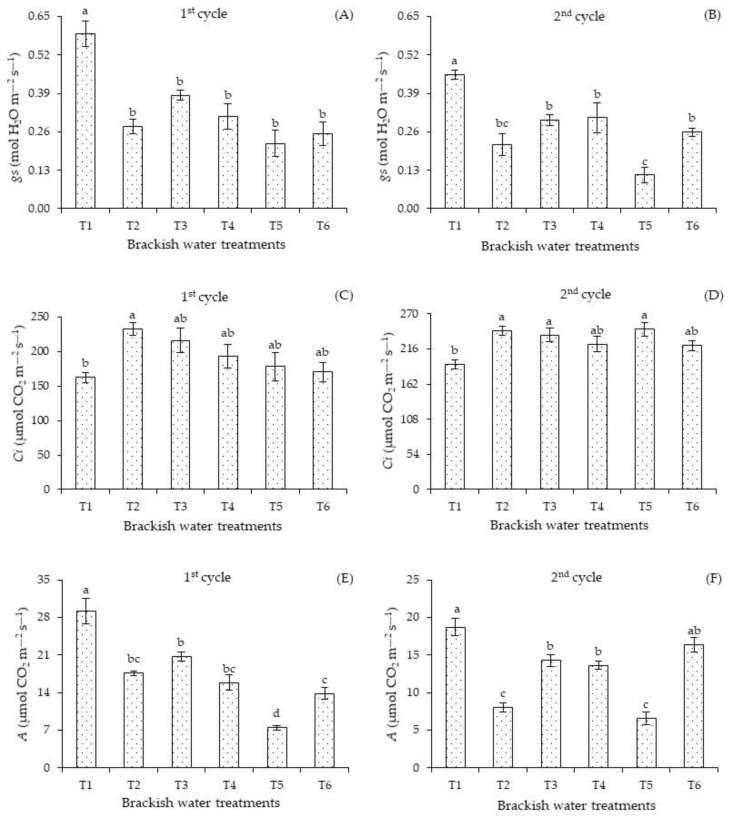
Stomatal conductance—*gs* (**A**,**B**), intercellular concentration of carbon dioxide—*Ci* (**C**,**D**), and photosynthesis—A (**E**,**F**) of *Passiflora edulis* Sims as a function of salt stress, varying the phenological stages in the first (240 DAT) and second (445 DAT) cycle. Standard error of the mean (*n* = 8); Means followed by different letters indicate a significant difference between treatments. T1—irrigation with low-salinity water during the entire cultivation cycle; salt stress in the following stages: T2—vegetative; T3—flowering; T4—fruiting; T5—vegetative and flowering; T6—vegetative and fruiting.

Stomatal conductance was negatively affected by irrigation with water of 4.0 dS m^−1^ in the two cultivation cycles, regardless of the plant development stage (Figure 5). In the first cycle (Figure 5A), the *gs* was superior in plants under T1 compared to plants subjected to salt stress, regardless of the irrigation strategy. In the second cycle (Figure 5B), plants under T1 also had the highest *gs*, differing significantly from those subjected to T2, T3, T4, T5, and T6. When comparing the stomatal conductance of plants irrigated with 4.0 dS m^−1^, it was observed that the salt stress applied in T5 resulted in the lowest value compared to the T3, T4, and T6 strategies.

Intercellular concentration of carbon dioxide (Figure 5C) under the treatment T1 in the first cycle did not differ significantly from the values found in T3, T4, T5, and T6. There were significant differences only between plants subjected to the T1 and T2 strategies. In the second cycle (Figure 5D), plants grown with 4.0 dS m^−1^ in T2, T3, and T5 obtained higher intercellular concentrations of carbon dioxide compared to treatment T1. However, there were no significant differences in the *Ci* of plants grown under T4 and T5 compared to the T1 strategy.

The photosynthesis of the plants was influenced by the irrigation strategies with brackish water (Figure 5E,F). In the first cycle (Figure 5E), irrigation with the treatment T1 promoted the highest photosynthesis compared to plants grown under other strategies. Continuous irrigation with 4.0 dS m^−1^ in the vegetative and flowering stages resulted in the lowest photosynthesis in both cycles.

In the second cycle (Figure 5F), the photosynthesis of plants under the treatment T5 did not differ significantly from the values of those receiving T1. This situation indicates a possible recovery of plants that received T2 and T4. The application of treatments T2 and T5 compromised the photosynthesis. In plants cultivated under irrigation with water of high electrical conductivity, the stomata usually close and therefore limit the entry of CO_2_ into the substomatal chamber.

**Figure 6:**
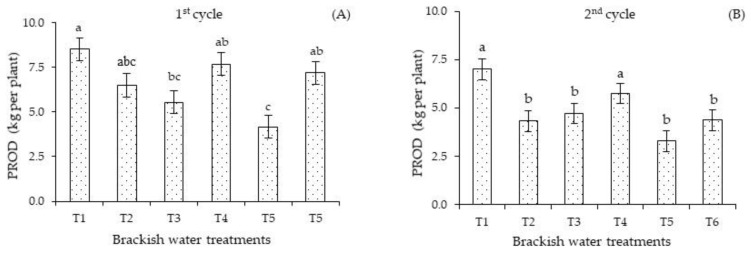
Production per plant—PROD of *Passiflora edulis* Sims as a function of salt stress, varying the phenological stages in the first (**A**) and second (**B**) cycles. Standard error of the mean (*n* = 8); Means followed by different letters indicate a significant difference between treatments. T1—irrigation with low-salinity water during the entire cultivation cycle; salt stress in the following stages: T2—vegetative; T3—flowering; T4—fruiting; T5—vegetative and flowering; T6—vegetative and fruiting.

The irrigation management strategies with brackish water influenced the production per plant of sour passion fruit. In the first cycle (Figure 6A), plants subjected to the T3 and T5 strategies attained the lowest PROD values (5.54 and 4.16 kg per plant). In the second cycle (Figure 6B), plants under strategies T2, T3, T5, and T6 (4.32, 4.72, 3.28, and 4.37 kg per plant) did not differ significantly among themselves and showed the lowest PROD values, on average 34.63% lower than that obtained with strategies T1 and T4 (7.00 and 5.75 kg per plant), which were statistically equal. The lowest PROD values observed in plants under strategy T5 reflect the changes observed in the gas exchange parameters, especially stomatal conductance, photosynthesis, and ion homeostasis. Another reason for the reduced production is the decrease in N, P, and K accumulations, which were considered insufficient for the nutrition of sour passion fruit [23], regardless of the irrigation strategy and the cultivation cycle.

The reduction in production per plant (Figure 6A,B) in the first cycle under strategies T3 and T5 and in the second cycle under T2, T3, T5, and T6 indicates that *Passiflora edulis* Sims is sensitive to salt stress during the vegetative and flowering stages. Plant sensitivity to salinity normally varies with the development stages.

In this research, the highest values of polar diameter of fruits in the first cycle were obtained in plants under the T1, T4, and T6 strategies, but did not differ significantly from those observed in plants grown with 4.0 dS m^−1^ water in T2 and T3. On the other hand, irrigation with high-salinity water in T5 resulted in lower values of fruit polar diameter compared to those found under T1, T4, and T6 strategies. In the second cycle, the fruits produced under the T2 and T5 strategies had lower polar diameter values compared to those of plants irrigated with ECw of 1.3 dS m^−1^ during the entire cycle. Regarding the equatorial diameter, passion fruit plants subjected to the T1, T2, T4, and T6 strategies had the highest values in the first cycle, while irrigation with ECw of 4.0 dS m^−1^ in T3 and T5 resulted in fruits with smaller equatorial diameter. In the second cycle, there was no significant effect of irrigation strategies with brackish water and K doses on the equatorial diameter of the fruits.

Regarding the chemical characteristics of the fruits, as highlighted by Lima et al. [3], irrigation with water of 4.0 dS m^−1^ in T5 and fertilization with 345 g K_2_O per plant per year increases the total titratable acidity and reduces the hydrogen potential and soluble solids in the passion fruit pulp, regardless of the adopted strategy. On the other hand, fertilization with 345 g K_2_O and irrigation with water of 4.0 dS m^−1^ in T3 increases the flavonoid contents and the ratio of soluble solids to total titratable acidity, while the salt stress in the fruiting stage promotes an increase in anthocyanin contents in the passion fruit pulp.

## 3. Discussion

In the semi-arid region of northeastern Brazil, the salt stress caused by the high concentrations of salts found in surface and subsurface water sources negatively interferes with plant growth and development. Salt stress induces osmotic, ionic, and oxidative stresses and changes in gene expression, hindering normal physiological processes and nutrient absorption and hence reducing plant production [28]. Thus, irrigation with high-salinity water at varying phenological stages is an alternative means to minimize the deleterious effects on plants through the maintenance of ionic homeostasis and modulation of physiology and through the reduction in negative impacts and physical-chemical attributes of the soil [29]. In addition, for performing osmoregulatory function, potassium also contributes to the ionic and osmotic homeostasis of plants.

Plants fertilized with 345 g K_2_O had higher N concentrations than those that received 207 g K_2_O under irrigation with 4.0 dS m^−1^ in the T3 and T6 strategies. Potassium plays a key role in the nitrogen metabolism of plants and can increase the synthesis of amino acids and proteins. Furthermore, this macronutrient contributes to the balance of cations and anions in the cytoplasm [30]. According to [31], plants that accumulate higher K contents tend to restrict the absorption and transport of toxic ions (Na^+^ and Cl^−^) from irrigation water.

This reduction in the second cycle could be related to the time of exposure of the plants to salt stress, which is considered one of the main factors responsible for the low N availability in the soil [32]. The results obtained for the N concentrations are lower than those found by [33], who evaluated the macronutrient concentrations of *Passiflora edulis f. flavicarpa Deg.* at the beginning of flowering under irrigation with saline water in soils with and without mineral fertilization and obtained the mean value of 50.81 g kg^−1^.

The increase in the leaf K concentrations is an important mechanism of plants under salt stress since this macronutrient plays a relevant role in stomatal regulation, especially under restricted conditions of water, and ensures the turgidity of guard cells through a reduction in the osmotic potential [34].

The phosphorus concentrations obtained in this study (1.91 to 2.53 g kg^−1^) are considered inadequate to meet the nutrient requirement of plants, i.e., they are below the range indicated by [23] (between 4 and 5 g kg^−1^). Carvalho et al. [35], in a study on the effects of potassium fertilization, irrigation depths, and times of the year on the concentrations of macronutrients, micronutrients, and Na^+^ of *Passiflora edulis f. flavicarpa*, also observed that the leaf concentrations of P and K in all seasons were below the range considered adequate for the cycle.

The higher reduction in the potassium concentrations of the second cultivation cycle could be related to the higher Na^+^ concentration compared to K^+^ in the root zone, with Na^+^ inhibiting the K^+^ uptake by plants due to the competition between Na^+^ and K^+^ at the uptake sites of the cell membranes. Sodium can replace potassium at the binding sites and, as a result, inhibit regular plant metabolism [13].

The leaf contents of N, P, and K, regardless of the phenological stage and the cultivation cycle, obtained in this study are below the range considered adequate by Malavolta et al. [23]. However, it is important to highlight that fertilization management was recommended by [25] and the plants at no stage of their cycle showed symptoms of nutritional deficiency. Soares et al. [26], in a study evaluating the effects of salinity on the early development of passion fruit, also found that potassium absorption was drastically reduced, even at low salinity levels. According to these authors, this reduction occurred due to the loss of capacity for selective ion absorption by the plasmalemma.

The reduction in the water content in plants subjected to salt stress (T1, T2, T3, T4, T5, and T6) is a result of the negative effect of osmotic stress on water availability in the soil and consequently reduced water uptake by the plants, which affects their general water status [36]. The reduction in the relative water content in the leaf blade in the different phenological stages influenced the gas exchange, limiting stomatal conductance and CO_2_ assimilation rate. Usually stomatal closure is a strategy to minimize water losses to the atmosphere and reduce excessive absorption of toxic ions (Na^+^ and Cl^−^).

Although the increase in electrolyte leakage occurred in plants under high salinity (4.0 dS m^−1^) compared to those that received ECw of 1.3 dS m^−1^, the values obtained were less than 50%, which is an indication that there was no significant damage to the cell membrane of the sour passion fruit, as the tissue is considered injured when the percentage of electrolyte leakage exceeds 50% [37].

Under salt stress conditions, plants normally lose water from their tissues, which can have rapid and significant effects on cell expansion and division, stomatal opening, and accumulation of abscisic acid [38]. On the other hand, salt stress induces the formation of reactive oxygen species (ROS), which cause oxidative damage and lipid peroxidation of the membrane, decreasing membrane fluidity and selectivity [4]. Thus, with the overproduction of ROS in the cells, the stability or integrity of the membrane is interrupted, resulting in electrolyte leakage in plants under stress conditions [36].

The partial stomatal closure in plants cultivated under salt stress decreases CO_2_ absorption and affects the functions of the photosynthetic apparatus [4]. The control of stomatal opening and closure is one of the adaptative mechanisms used to avoid the loss of cell turgor due to the limited water supply [39]. As observed in this study, Lima et al. [40] found that irrigation with water of 3.2 dS m^−1^ limited the stomatal conductance of plants subjected to salt stress in the vegetative, vegetative/flowering, flowering, fruiting, and vegetative/fruiting stages. According to these authors, the lower values of stomatal conductance in plants under salt stress occur due to reductions in leaf turgor and atmospheric vapor pressure, which are ways to reduce the delay between water absorption by roots and transpiration, consequently leading to partial closure of the stomata to avoid excessive dehydration of the guard cells.

The increase in the *Ci* of plants grown under salt stress indicates damage to the photosynthetic apparatus, e.g., decrease in the carboxylation efficiency of RuBisCO, caused mainly by salt accumulation in the leaf tissues [41].

The increase in the intercellular concentration of carbon dioxide in plants subjected to T5 did not influence photosynthesis, suggesting that this process was inhibited by the action of factors of non-stomatal origin. The non-stomatal regulation induced by salt stress is related to the activities of the photosynthetic enzymes of the Calvin cycle, disturbances in chlorophyll synthesis, and damage to the photosynthetic apparatus [42].

The reduction in the concentration of CO_2_ in the substomatal spaces is reflected in the rate of CO_2_ assimilation. Another factor contributing to the decrease in CO_2_ assimilation is the inhibition of the RuBisCO enzyme activity, which prevents the conversion of absorbed CO_2_ into photoassimilates. In many cycles, notably, salt stress interferes with traits of gas exchange under moderate to severe levels of salinity, thus reducing stomatal conductance and the rate of CO_2_ assimilation due to the limitations that occur in the total water potential caused by excess salts [43].

However, most plants, especially those of economic importance, are more sensitive to salinity during the early phenological stages [16], which may have contributed to the observed lower production per plant. Further, the higher energy expenditure to maintain the metabolic activities under salt stress conditions may have caused the formation of fruits with lower mass [44], thus justifying the reduced PROD in plants under these strategies. The reduction in production mainly in plants subjected to salt stress in the flowering and flowering/fruiting stages stands out as a challenge for irrigated cultivation, since the water requirement in these stages is greater when compared to the vegetative stage; with this, more salts are incorporated into the soil, compromising the production of plants [45].

Unlike the results obtained in this study, Souto et al. [31] concluded that there was an increase in the photosynthesis of yellow passion fruit plants grafted on *P. cincinnata*, but this was not reflected in fruit yield. In the present research, the reduction in photosynthesis observed in plants subjected to salt stress in T5 in both crop cycles decreased production per plant. Soares et al. [16], in a study with cotton genotypes, observed that the successive application of saline water in the flowering and boll formation stages caused a drastic reduction in the physiological aspects of the cycle, with recovery of the plants after the interruption of stress.

In general, it is possible to observe that sour passion fruit showed contrasting responses in NPK accumulation, physiology, and production as a function of the different phenological stages and production cycles, proving that the sensitivity and/or tolerance of plants depends on several factors such as intensity and duration of stress, irrigation and fertilization management practices, and soil and climatic conditions of the region.

## 4. Materials and Methods

### 4.1. Characterization of Study

The research was carried out in the field at the Center of Agricultural Sciences and Technology (CCTA), in Pombal, PB, located at 06°48′50″ S, 37°56′31″ W, at an altitude of 190 m.

### 4.2. Details of Sources of Variation and Period of Application of Treatments

**Figure 7:**
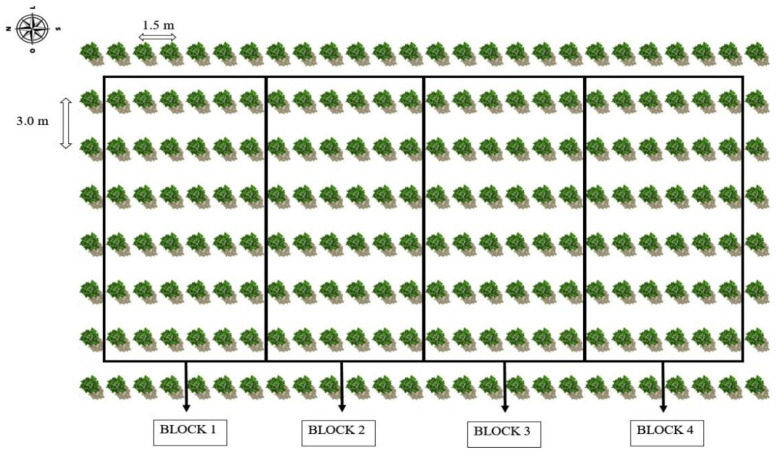
Layout of the experimental area.

The experiment consisted of six irrigation strategies with brackish water—ISBW (T1—irrigation with low-salinity water during the entire cultivation cycle as a control; irrigation with high-salinity water at the respective stages: T2—vegetative; T3—flowering; T4—fruiting; at the successive stages, vegetative and flowering—T5; vegetative and fruiting—T6 and two potassium levels corresponding to 207 and 345 g of K_2_O per plant per year of the potassium recommendation [25], distributed in randomized blocks in a 6 × 2 factorial arrangement with four replications, with each plot consisting of three usable plants. The experimental layout is shown in Figure 7. The area had a row of plants externally to the usable plots.

Two salinity levels of irrigation water were used in the experiment, one corresponding to moderate salinity (1.3 dS m^−1^—T1) and the other to high electrical conductivity (4.0 dS m^−1^—T2, T3, T4, T5, and T6), applied during the following cycle development stages in the first cycle: T1—irrigation with low-salinity water during the entire cultivation cycle (1–253 days after transplanting—DAT); irrigation with high-salinity water in T2—from transplanting until the formation of the floral primordia (50–113 DAT); T3—from the formation of the floral primordia to the total development of the flower bud (anthesis) (114–198 DAT); T4—from the fecundation of the flower bud to the formation of fruits with interspersed yellow spots (199–253 DAT); T5—in the vegetative and flowering stages (50–198 DAT); T6—in the vegetative (50–113 DAT) and fruiting stages (199–253 DAT). The procedure was similar in the second cultivation cycle, T1—irrigation with low-salinity water during the entire cultivation cycle (254–475 DAT); salt stress in T2 (254–340 DAT); T3—flowering (341–360 DAT); T4—fruiting (361–475 DAT); T5—vegetative and flowering stages (254–360 DAT); T6—vegetative (254–340 DAT) and fruiting stages (361–475 DAT).

### 4.3. Crop Management

In this research, the BRS GA1 genotype was used, as it has a great potential for exploitation in Brazilian orchards due to its high yield (42 t ha^−1^) and tolerance to Anthracnose. The seedlings were produced by sowing two seeds in plastic bags filled with a substrate composed of soil, sand, and cattle manure in the ratio of 84:15:1 (*v*/*v*). From the moment the plants started to produce tendrils, transplanting to the field was carried out (61 DAS).

Before transplanting the seedlings, plowing and harrowing were carried out in the area. The sour passion fruit was cultivated in an Entisol, with the following physical and chemical properties determined according to [46]: Hydrogen potential (1:2.5 soil/water) = 7.82, organic matter = 0.81 dag kg^−1^, P = 10.60 mg kg^−1^, P = 10.60 mg kg^−1^; K^+^, Na^+^, Ca^2+^, Mg^2+^ and Al^3+^ + H^+^ equivalent to 0.30, 0.81, 2.44, 1.81, and 0 cmol_c_ kg^−1^, respectively; Particle-size fraction: Sand, Silt and Clay = 820.90, 170.10, and 9.00 g kg^−1^, respectively; Moisture content (dag kg^−1^) at field capacity (33.42 kPa) and permanent wilting point (1519.5 kPa) = 12.87 and 5.29, respectively.

The sour passion fruit plant was cultivated in holes measuring 40 cm × 40 cm × 40 cm. In the basal fertilization, 20 L of bovine manure and 50 g of single superphosphate were used, following the recommendation of [25]. Nitrogen and potassium were applied in the top dressing, using urea and potassium chloride, respectively. Plants subjected to the dose of 345 g K_2_O per plant received 65 g in the vegetative stage and 280 g during the flowering and fruiting stages. The other plants received 39 and 168 g K_2_O per plant in the same stages of development.

Every 15 days after plant emergence the micronutrients were applied using 1 g L^−1^ of Dripsol Micro^®^ (Candeias, Bahia, Brazil), containing 1.1, 0.85, 0.5, 3.4, 3.2, 0.05, and 4.2% of Mg^2+^, B, Cu, Fe, Mn, Mo, and Zn, respectively. The applications were carried out through the leaves. The plants were cultivated with a spacing of 3 m × 3 m, adopting the vertical trellis system at a height of 1.80 m. A nylon ribbon was used to guide the plants. When plants exceeded the trellis height by 10 cm, the apical bud was cut to induce the formation of secondary branches. After the production of the secondary branches, two were conducted, one on each side up to a length of 1.5 m, and later were conducted up to 30 cm from the ground level. Tendrils and unwanted branches were eliminated throughout the crop cycle to promote the full development of the crop.

At 254 DAT the second production cycle began. At this time, the canopy was pruned [3]. This procedure reduces the problems caused by pests and diseases, improves the phytosanitary status of plants, and facilitates crop management, especially fertilization and irrigation. The pruning of tertiary and quaternary branches was performed at 40 cm from the wire.

The 1.3 dS m^−1^ water was obtained from a CCTA/UFCG well, with Ca^2+^, Mg^2+^, Na^+^, K^+^, HCO_3_^−^, CO_3_^2−^, and Cl^−^ concentrations of 0.85, 0.40, 5.81, 0.40, 5.09, 0, and 4.07 mmol_c_ L^−1^, respectively, and hydrogen potential of 6.69. Water with electrical conductivity of 4.0 dS m^−1^ was prepared by adding NaCl, based on the relationship between ECw and salt concentration [47].

Brackish water irrigation management began at 50 DAT. A drip irrigation system was adopted, using 32-mm-diameter PVC tubes in the main line and 16-mm-diameter low-density polyethylene tubes in the lateral lines, with emitters operating at a flow rate of 10 L h^−1^. Each plant had two pressure-compensating drippers (model GA 10 Grapa), each at 15 cm from the stem. The plants were irrigated daily at 7:00 a.m. by supplying water according to the strategy adopted. The irrigation depth was estimated based on the crop evapotranspiration [48], obtained using Equation (1):

ETc = ETo × Kc (1)

where:

ETc—crop evapotranspiration, mm day^−1^;

ETo—reference evapotranspiration of Penman-Monteith, mm day^−1^; and

Kc—crop coefficient, dimensionless.

Climatic data obtained from the Meteorological Station were used to quantify the reference evapotranspiration (ETo) using the crop coefficients recommended by [49]. It is important to point out that irrigation with water of high and low electrical conductivity, varying the phenological stages, reduces salt stress in plants and impacts on the salt accumulation in soils. Additionally, at 30-day intervals, a leaching fraction of 0.15 was applied to reduce the salt content in the soil. The water depths applied in the different irrigation strategies are presented in Table 3.

### 4.4. Variables Analyzed

The mineral composition (N, P, K) was evaluated by selecting the fourth leaf from the apex of the intermediate branches of each experimental unit according to [33]. The collection was performed during the transition from flowering to fruiting in the first (199 DAT) and second (361 DAT) production cycles. After drying, the samples were ground and subjected to chemical analyses according to the methodology of [23]. The P and K concentrations were determined by nitric acid digestion, whereas the N concentration was determined by sulfuric acid digestion.

The relative water content (RWC), electrolyte leakage (EL) in the leaf blade, and gas exchange were also determined at 199 DAT (1st cycle) and 361 DAT (2nd cycle). The relative water content (RWC) in the leaf blade was evaluated using 8 leaf discs with areas of 113 mm^2^, collected from leaves located in the middle third of the secondary branches. Immediately after, the discs were weighed to obtain the fresh mass (FM). The samples were placed in plastic bags, immersed in 50 mL of distilled water, and stored for 24 h. After this period, the excess water was removed with paper towels, and the samples were weighed to obtain the turgid mass (TM). Subsequently, the samples were dried in the oven (temperature ≈ 65 °C ± 3 °C, until reaching constant weight) to obtain the dry mass (DM). The RWC was determined according to [50] using Equation (2):

(2) $$\mathrm{RWC}=\frac{\left(\mathrm{FM}-\mathrm{DM}\right)}{\left(\mathrm{TM}-\mathrm{DM}\right)}\;\times \;100$$

where:

RWC—relative water content (%);

FM—leaf fresh mass (g);

TM—leaf turgid mass (g); and

DM—leaf dry mass (g).

To determine electrolyte leakage in the leaf blade, leaves were collected from the middle third of the secondary branches; 8 leaf discs with areas of 113 mm^2^ were immediately removed, washed with distilled water to eliminate other electrolytes adhered to the leaves, and then placed in a beaker with 50 mL of double-distilled water, which was closed with aluminum foil. The samples remained at a temperature of 25 °C for 24 h and then the initial electrical conductivity (Ci) was measured; subsequently, the beakers were taken to an oven with forced air circulation and subjected to a temperature of 80 °C for 150 min and then cooled to determine the final electrical conductivity (Cf). Electrolyte leakage in the leaf blade was obtained according to [51] using Equation (3):

(3) $$\%\mathrm{EL}=\frac{\mathrm{Ci}}{\mathrm{Cf}\;}\;\times \;100\;$$

where:

%EL—electrolyte leakage in the leaf blade;

Ci—initial electrical conductivity (dS m^−1^); and

Cf—final electrical conductivity (dS m^−1^).

Gas exchange was evaluated using an intermediate and intact leaf of the productive branch to determine stomatal conductance (gs-mol H_2_O m^−2^ s^−1^), photosynthesis (A) (μmol CO_2_ m^−2^ s^−1^), and intercellular concentration of carbon dioxide (Ci) (μmol CO_2_ m^−2^ s^−1^) using the portable photosynthesis meter “LCPro+” from ADC BioScientific Ltd. (Hoddesdon, England). The readings were performed from 7:00 to 10:00 a.m. using the third fully expanded leaf counting from the apical bud under natural conditions of air temperature, CO_2_ concentration, and using an artificial radiation source established through the photosynthetic light response curve and determining the photosynthetic light saturation point [52].

Mature fruits (with a yellow peel color) were harvested from 199 to 253 DAT in the first cycle and from 361 to 445 DAT in the second cycle. The first cycle comprised the period from transplanting to the end of the harvest (1–253 DAT). At the end of harvest, a cleaning pruning was carried out to renew productive branches, eliminating dead, old, diseased and/or unproductive branches, reducing the problems caused by pests and diseases and improving the phytosanitary state of the plants. The second cycle began after pruning, keeping the same plants used in the first production cycle (254–475 DAT). The cultivation of passion fruit in the second production cycle had the purpose of validating the changes that occurred in the first cycle in the accumulation of NPK, in physiology, and production, considering that the responses of plants to stress vary according to the time of year and the irrigation management, fertilization, and climatic conditions. After harvest, the production per plant (PROD, kg per plant) was determined and fruits harvested per treatment were counted, allowing the calculation of average fruit weight.

### 4.5. Statistical Procedures

The data obtained were evaluated through analysis of variance by the F test after the data normality test (Shapiro-Wilk). Then, an analysis of variance was performed, with the Tukey test (*p* ≤ 0.05) used for irrigation strategies and K doses. These analyses were performed using the statistical software SISVAR ESAL version 5.7 [53].

## 5. Conclusions

Relative water content, stomatal conductance, and photosynthesis were reduced by salt stress during the first cultivation cycle. However, in the second cycle, irrigation with 4.0 dS m^−1^ water in the fruiting stage and successively in the vegetative and fruiting stages resulted in the highest production per plant and in elevation of photosynthesis, respectively. In the present study, the concentrations of N, P, and K in passion fruit leaves are below the optimal levels established in the literature, regardless of the development stage and crop cycle. Changes in ionic homeostasis by inhibition of NPK absorption caused a reduction in the production of passion fruit under the semi-arid conditions of Northeast Brazil, leading to a yield lower than the production potential of the BRS GA1 cultivar. Despite the importance of potassium in osmoregulation and its role in various physiological processes, such as stomatal regulation, photosynthetic fixation of CO_2_, and transport and use of photoassimilates, in the present study, no significant effects of potassium doses were observed on the relative water content and electrolyte leakage in the leaf blade as well as in the gas exchange of sour passion fruit in both cultivation cycles. The hypothesis formulated was confirmed only for potassium concentrations, as fertilization with the recommended dose of 100% increased the levels of K in plants cultivated under irrigation water salinity of 1.3 dS m^−1^ throughout the growth cycle and under water salinity of 4.0 dS m^−1^ in the fruiting and vegetative/fruiting stages in the first cycle and in the vegetative stage in the second cycle. To expand knowledge about *Passiflora edulis* Sims under salt stress, future studies should elucidate the effects of salinity on the activity of antioxidant enzymes and the accumulation of organic solutes and their role in osmotic adjustment, in addition to ionic homeostasis through the determination of levels of macro and micronutrients and the accumulation of sodium and chloride in passion fruit plants.

## Figures and Tables

**Table 1:** Summary of the analysis of variance referring to the leaf concentrations of nitrogen (N), phosphorus (P), and potassium (K) in *Passiflora edulis* Sims under brackish water irrigation strategies (ISBW) and potassium fertilization (KD).

	Mean Squares					
	ISBW	KD	ISBW × KD	Blocks	Residual	CV (%)
	1st cycle					
N	81.35 **	22.77 *	41.48 *	6.22 ^ns^	6.30	13.67
P	0.42 *	0.34 ^ns^	0.25 ^ns^	0.49 *	0.16	18.26
K	112.46 **	100.31 **	10.75 *	9.00 ^ns^	4.55	15.35
	2nd cycle					
N	26.14 **	3.34 ^ns^	15.26 *	1.65 ^ns^	4.09	14.21
P	0.15 ^ns^	0.16 ^ns^	0.02 ^ns^	0.07 ^ns^	0.05	14.71
K	102.09 **	78.23 *	19.48 *	8.14 ^ns^	5.10	24.77
DF	5	1	5	3	33	-

DF—degree of freedom; CV (%)—coefficient of variation; * significant at 0.05 probability level; ** significant at 0.01 probability level; ^ns^ not significant.

**Table 2:** Analysis of variance for relative water content (RWC), electrolyte leakage (%EL), stomatal conductance (*gs*), intercellular concentration of carbon dioxide (*Ci*), photosynthesis (*A*), and production per plant (PROD) in *Passiflora edulis* Sims under brackish water irrigation strategies (ISBW) (ISBW) and potassium fertilization (KD).

	Mean Squares					
	ISBW	KD	ISBW × KD	Blocks	Residual	CV (%)
	1st cycle					
RWC	969.94 **	91.52 ^ns^	52.08 ^ns^	22.16 ^ns^	82.43	13.29
%EL	52.92 **	12.56 ^ns^	5.74 ^ns^	2.77 ^ns^	4.73	14.72
*gs*	0.14 **	0.01 ^ns^	0.004 ^ns^	0.004 ^ns^	0.01	32.76
*Ci*	5944.07 *	0.02 ^ns^	3059.97 ^ns^	213.57 ^ns^	1787.48	22.04
*A*	418.15 **	9.62 ^ns^	21.38 ^ns^	5.10 ^ns^	12.71	20.46
PROD	1,989,844.2 **	13,612,052.5 ^ns^	5,063,282.6 ^ns^	1,225,254.3 ^ns^	2866,438.2	25.63
	2nd cycle					
RWC	764.30 **	186.48 ^ns^	18.42 ^ns^	69.36 ^ns^	48.55	11.06
%EL	149.28 **	14.64	18.00	12.67	8.97	14.67
*gs*	0.10 **	0.004 ^ns^	0.005 ^ns^	0.01 ^ns^	0.006	28.91
*Ci*	3213.20 *	74.32 ^ns^	135.61 ^ns^	1161.56 ^ns^	763.06 ^ns^	12.15
*A*	177.71 **	2.55 ^ns^	8.27 ^ns^	15.46 ^ns^	4.41 ^ns^	16.26
PROD	1,989,844.2 **	13,612,052.5 ^ns^	5,063,282.6 ^ns^	1,225,254.3 ^ns^	2,866,438.2	27.48
DF	5	1	5	3	33	-

DF—degree of freedom; CV (%)—coefficient of variation; * significant at 0.05 probability level; ** significant at 0.01 probability level; ^ns^ not significant.

**Table 3:** Water depth values applied to BRS GA1 yellow passion fruit in different strategies of irrigation with brackish water in the phenological stages.

ISBW	1st Cycle		
	DAT	Water Depth (mm)	
		1.3 dS m^−1^	4.0 dS m^−1^
T1	1–253	1256	0
T2	50–113	877	253
T3	114–198	847	273
T4	199–253	1079	119
T5	50–198	468	525
T6	50–113/199–253	699	371
	2nd cycle		
T1	254–475	1043	0
T2	254–340	628	277
T3	341–360	924	79
T4	361–475	533	340
T5	254–360	510	356
T6	254–340/361–475	119	616

ISBW—irrigation strategies with brackish water; T1—irrigation with low-salinity water during the entire cultivation cycle; salt stress in the following stages: T2—vegetative; T3—flowering; T4—fruiting; T5—vegetative and flowering; T6—vegetative and fruiting.
